# Pregnancy-Associated Cancer: A Systematic Review and Meta-Analysis

**DOI:** 10.1016/j.mayocpiqo.2024.02.002

**Published:** 2024-03-16

**Authors:** Ben Walters, India Midwinter, Carolyn A. Chew-Graham, Kelvin P. Jordan, Garima Sharma, Lucy C. Chappell, Emma J. Crosbie, Purvi Parwani, Mamas A. Mamas, Pensée Wu

**Affiliations:** a Academic Department of Obstetrics and Gynaecology, Royal Stoke Hospital, Stoke-on-Trent, United Kingdom; b School of Medicine, Faculty of Medicine and Health Sciences, Keele University, Staffordshire, United Kingdom; c Division of Cardiology, Johns Hopkins Ciccarone Center for Prevention of Cardiovascular Diseases, Johns Hopkins University School of Medicine, Baltimore, MD; d School of Life Course Sciences, King’s College London, London, United Kingdom; e Department of Obstetrics and Gynaecology, St Mary’s Hospital, Manchester University NHS Foundation Trust, Manchester Academic Health Science Centre, Manchester, United Kingdom; f Division of Cancer Sciences, School of Medical Sciences, Faculty of Biology, Medicine and Health, University of Manchester, Manchester, United Kingdom; g Division of Cardiology, Department of Medicine, Loma Linda University Health, Loma Linda, CA; h Keele Cardiovascular Research Group, Centre for Prognosis Research, School of Medicine, Faculty of Medicine and Health Sciences, Keele University, Staffordshire, United Kingdom; i Academic Department of Cardiology, Royal Stoke Hospital, Stoke-on-Trent, United Kingdom; j Department of Obstetrics and Gynecology, College of Medicine, National Cheng Kung University, Tainan, Taiwan

## Abstract

This study aimed to systematically evaluate and quantify the risk of adverse maternal and neonatal outcomes in patients with pregnancy-associated cancer (PAC). This study was conducted from February 13, 2021, through July 24, 2023. A systematic search of MEDLINE, Embase, Web of Science Core Collection, Cochrane Database of Systematic Reviews, and Cochrane Central Register of Controlled Trials was conducted to identify studies reporting outcomes for patients with PAC. The study was registered on PROSPERO. Two reviewers independently conducted screening, data extraction, and quality assessment. The associations were quantified using random-effects meta-analysis. The initial search produced 29,401 titles and abstracts, after which 147 unique full-text articles were screened, of which 22 articles with 59,190 pregnancies with PAC from 70,097,167 births were included in the meta-analysis. Women with PAC were at significantly increased risk of cesarean deliveries (risk ratio [RR], 1.58; 95% CI, 1.31-1.89), preterm birth (RR, 3.07; 95% CI, 2.37-3.98), venous thromboembolism (RR, 6.76; 95% CI, 5.08-8.99), and maternal death (RR, 41.58; 95% CI, 20.38-84.83). The only outcome with reduced risk was instrumental mode of delivery (RR, 0.67; 95% CI, 0.52-0.87). Pregnancy-associated cancer increases risk of adverse outcomes, including a 7-fold risk of venous thromboembolism and a 42-fold risk of maternal death. Further research is required to better understand the mechanisms leading to these adverse outcomes, especially for women who are not diagnosed until the postpartum period. Affected women should have counseling regarding their increased risk of adverse outcomes.

**Article Highlights**

• Women with pregnancy-associated cancer have double the risk of pregnancy, fetal, and neonatal complications.
• Pregnancy-associated cancer is associated with a 42-fold increased risk of maternal mortality.
• Breast cancer had the highest risk of preterm birth and low-birth weight.

Cancer is a leading cause of death globally. Pregnancy-associated cancer (PAC) occurs in 1 in every 1000 pregnancies.^1^ The definition of PAC varies across the literature, but the general consensus is that PAC refers to cancer that is diagnosed either during pregnancy or within 1 year of delivery. There has been a gradual increase in the incidence of pregnancies complicated by PAC over recent decades, which mirrors the increased incidence of breast, thyroid, and skin cancers in women of reproductive age.^2^, ^3^, ^4^, ^5^, ^6^ One-quarter of cases of PAC are identified during pregnancy, with the rest diagnosed up to a year postpartum.^1^ The most frequently occurring cancers during pregnancy are cervical, breast, melanoma, lymphoma, and leukemia.^7^ These histologic types are among the most frequent encountered in nonpregnant women of similar ages.^8^

Pregnancy-associated cancer has been reported by observational studies to be associated with adverse pregnancy outcomes, including premature rupture of membranes, preeclampsia, obstetric hemorrhage, and cesarean deliveries.^2^^,^^9^, ^10^, ^11^ Although not absolutely contraindicated, radiotherapy is used with caution during pregnancy, and treatment of PAC is usually limited to operation or chemotherapy. Although operation can be performed throughout pregnancy, it is usually limited to the second trimester where possible, to minimize the risk of miscarriage and preterm birth.^12^ Some chemotherapeutic agents can be administered safely during the second and third trimesters,^13^^,^^14^ whereas its use in the first trimester is avoided owing to the high likelihood of embryonic and fetal toxicity. During the first 10 days after conception, chemotherapy may lead to death of the embryo.^15^ Furthermore, cytotoxic medication administered during the first trimester has a 7%-19% risk of congenital malformations and spontaneous miscarriage.^16^ After the first trimester, cytotoxic medication is associated with a 11% risk of preterm delivery and low-birth weight.^17^^,^^18^ Previous studies have reported little effect of cytotoxic agents on the long-term outcomes of the child.^15^

Patients with PAC need to understand their risks of complications and be supported in their decision-making. However, this is challenging owing to limited evidence. The existing literature consists mainly of single-center and cohort studies, which are rarely generalizable to the entire population. To our knowledge, there are no previous published meta-analyses focusing on maternal and fetal outcomes in patients with PAC.

## Patients and Methods

### Eligibility Criteria and Information Sources

The study protocol was registered on the PROSPERO database of systematic reviews (Protocol number CRD42021287895).^19^ The 2020 Preferred Reporting Items for Systematic Reviews and Meta-Analyses and MOOSE guidelines were followed.^20^

Studies for inclusion were all study designs in which obstetric, maternal, or fetal outcomes associated with coexisting PAC were reported. Pregnancy-associated cancer was defined as cancer diagnosed in the 9 months before birth or in the year after birth of the child. We focused on studies on human participants and excluded animal or laboratory studies and case reports. Conference proceedings and case reports were excluded. Studies had to consider one of the following outcomes: pregnancy complications (gestational diabetes; hypertensive disorders of pregnancy including pre-eclampsia, eclampsia, and gestational hypertension; placenta previa; premature rupture of membranes; preterm birth; venous thromboembolism; and maternal death); intrapartum or peripartum complications (cesarean delivery, induction of labor, instrumental delivery, major puerperal infection, placental abruption, and postpartum hemorrhage); or fetal or neonatal complications (congenital malformations, low-birth weight, fetal growth restriction, intrauterine death, and neonatal and infant death).

### Search Strategy

This study was conducted from February 13, 2021, through July 24, 2023. Comprehensive searches in MEDLINE, Embase, Web of Science, and the Cochrane Library were conducted to identify studies between inception and February 13, 2021. The searches were rechecked by a clinical effectiveness librarian on January 12, 2023, to ensure complete coverage of all available literature. The search terms, including their synonyms, were (*pregnancy*) AND (*cancer*) AND (*antepartum haemorrhage*, *caesarean section*, *congenital malformations*, *fetal death*, *fetal distress*, *fetal growth restriction*, *gestational diabetes*, *instrumental delivery*, *low birth weight*, *perinatal death* or *mortality*, *placental abruption*, *placenta praevia*, *preeclampsia*, *pregnancy induced hypertension*, *preterm labo*ur, *postpartum haemorrhage*, *venous thromboembolism*, and *maternal death*) (Supplemental Table 1, available online at http://www.mcpiqojournal.org). There were no restrictions on language or date.

### Study Selection

Two reviewers (B.W. and I.M.) independently screened all titles and abstracts. Full texts of studies potentially meeting the inclusion criteria were then independently reviewed by B.W. and I.M. to assess eligibility for inclusion. Conflicts between reviewers were resolved by discussion with a third reviewer (P.W.). Manual searching for additional articles was also conducted by reviewing the bibliography of the screened full texts.

### Data Extraction, Synthesis, and Analysis

Data were independently extracted by both B.W. and I.M. We used RevMan version 5 (Cochrane Collaboration) to conduct a random-effects meta-analysis. We used random effects because studies were conducted across a varying array of settings and populations, and therefore, the analysis required consideration of the heterogeneity of these studies. Owing to the variability in how data were reported across the studies, we chose to meta-analyze the reported number of events of each outcome, rather than odds, or adjusted odds, ratios presented by the studies. Heterogeneity was assessed using the *I*^2^ statistic. Funnel plots were used to assess for publication bias in the case of an analysis in which there were more than 10 studies.

### Assessment of Risk of Bias

Risk of bias of selected studies was completed using the Brazzelli risk-of-bias tool for nonrandomized studies^21^ and the Oxford Centre for Evidence-Based Medicine grading system.^22^ Biases were independently assessed and compared by both B.W. and I.M., with conflicts resolved by discussion. Where conflicts arose, which could not be resolved by discussion, a third reviewer (P.W.) provided a final decision.

## Results

### Study Selection

**Figure 1:**
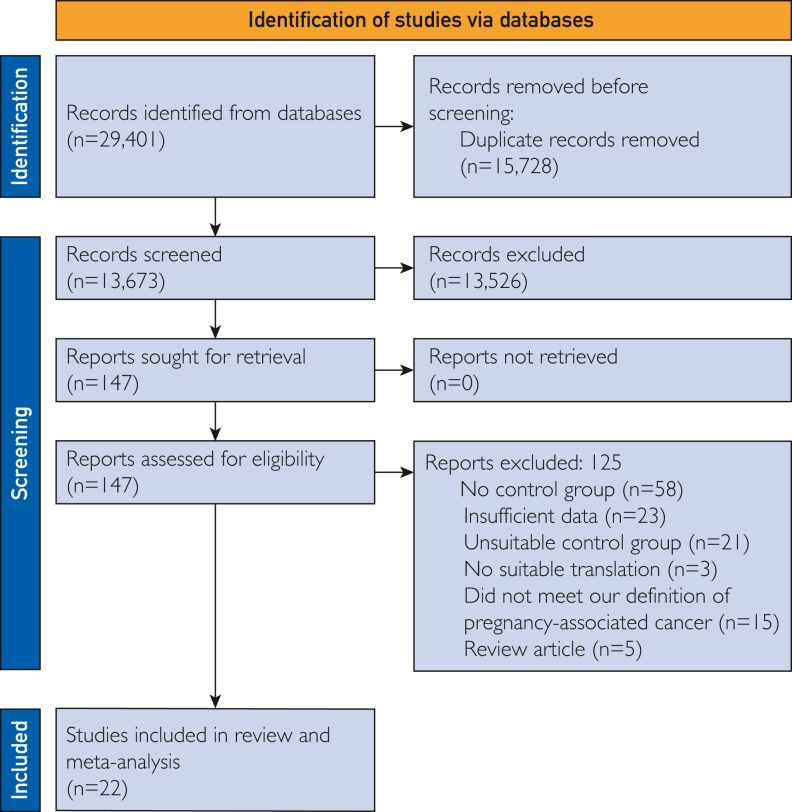
Preferred Reporting Items for Systematic Reviews and Meta-Analyses flowchart.

Initial database search identified 29,401 titles and abstracts for screening (Figure 1). After removal of 15,728 duplicates and screening, 22 studies met the eligibility criteria. These 22 studies reported 70,097,167 births, including 59,190 pregnancies with PAC, which equates to an overall incidence of 84.44 per 100,000 births. From the data available, 44,262 cancers were diagnosed during pregnancy and 5722 were diagnosed after delivery.

### Study Characteristics

The characteristics of the included studies are summarized in Supplemental Table 2 (available online at http://www.mcpiqojournal.org). All except 2 were retrospective in nature. Although patient-level data were unavailable, the mean of the reported mean ages of women with PAC was 30 years (range, 15-49 years) across the 12 publications that presented sufficient information to calculate.

Studies were drawn from a variety of databases, primarily the US National Inpatient Sample database (8 studies), California Office of Planning and Development database (2 studies), Scandinavian Health Registries (6 studies), New South Wales Population Databases (3 studies), and local center-level databases. Ten studies reported on multiple sites of cancer, whereas 13 studies reported on individual sites (breast cancer, cervical cancer, lymphoma, melanoma, ovarian cancer, colorectal cancer, and choriocarcinoma) only. None of the studies were restricted to first pregnancies or live births. Six studies reported on 3 overlapping populations (Abenhaim et al^23^ and Maor et al^24^ on breast cancer; and Greiber et al,^25^^,^^26^ Ma et al,^3^ and Wu et al^2^ on multiple cancers) but focusing on different outcomes mostly. For the outcomes which overlapped (preterm birth, postpartum hemorrhage, intrauterine death, and cesarean delivery), we used data from Wu et al,^2^ which had a larger study population.

Some key definitions used within this review were inconsistently defined across studies. Of the 11 studies that had low-birth weight as an outcome, low-birth weight was defined as birth weight less than 2500 g in 5 studies,^27^, ^28^, ^29^, ^30^, ^31^ less than the 10th percentile for gestational age in 4 studies,^9^^,^^32^, ^33^, ^34^ less than or equal to 2 standard deviations of the expected birth weight in 1 study,^26^ and by using the recorded International Classification of Diseases, Ninth Revision, code in 1 study.^35^ All 5 studies with the fetal growth restriction outcome used the recorded International Classification of Diseases, Ninth Revision codes to define their outcome. Of the 16 studies that assessed intrauterine death, 4 studies defined this as death occurring at 20 weeks or more of gestation,^30^, ^31^, ^32^^,^^34^ 3 studies as death at 28 weeks or more of gestation,^27^, ^28^, ^29^ 2 studies as death at 28 weeks or more of gestation for older data (before 2004^26^ or 2009^33^), and death at 22 weeks or more for more recent data (from 2004^26^ or 2009^33^), and 1 study as death at 22 weeks or more of gestation.^9^

### Risk of Bias of Included Studies

Supplemental Figure 1 (available online at http://www.mcpiqojournal.org) reports the quality assessment of included studies. All studies were either case-control or cohort studies, which were assessed as Oxford Centre for Evidence-Based Medicine grades III-IV.^22^ There were 19 studies that investigated outcomes of patients using national or multinational databases. There was a low to medium risk of bias in most areas across all studies. Key areas of high risk of bias were lack of prospective and blinded data collection.

### Synthesis of Results

#### PAC and Pregnancy Complications

**Figure 2:**
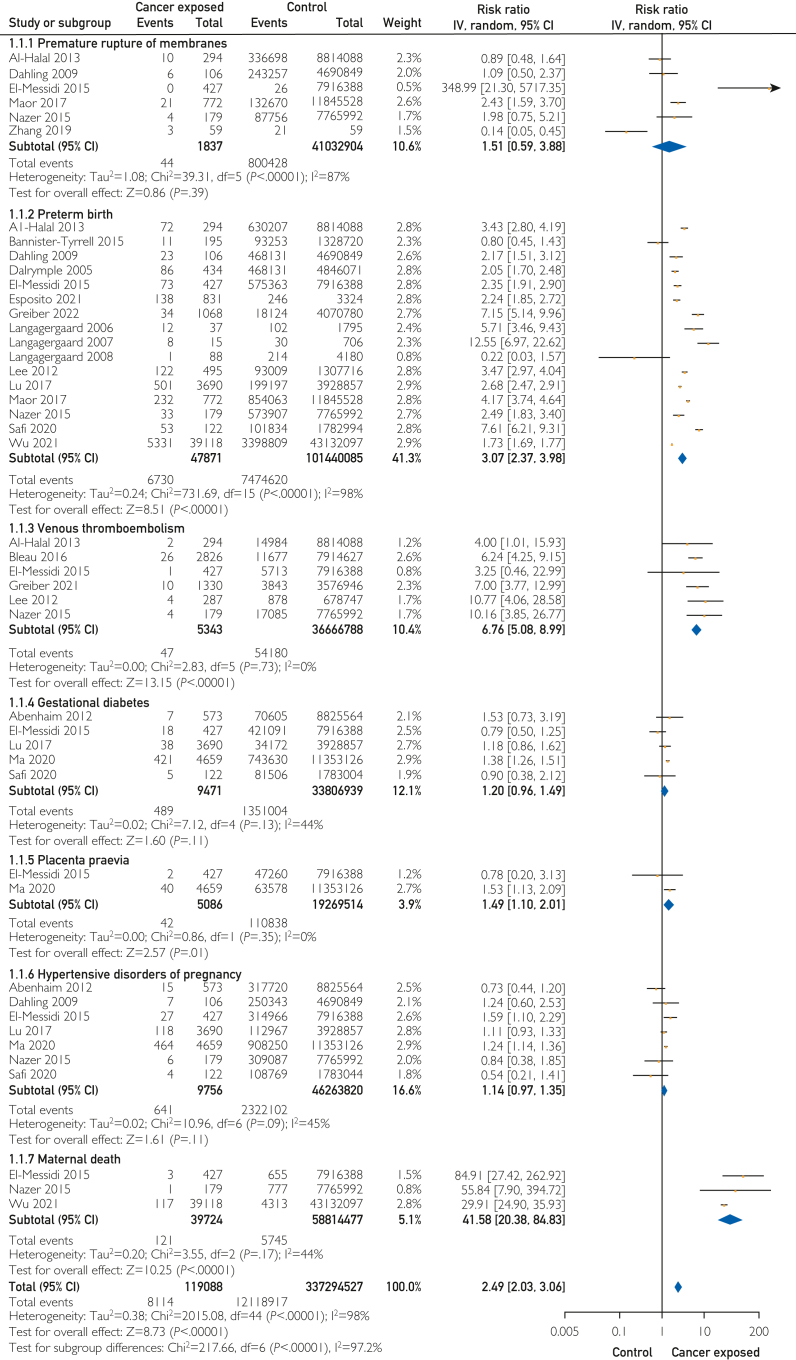
Pregnancy-associated cancer and pregnancy complications.

As reported in Figure 2, there was an almost 7-fold increased risk of venous thromboembolism (6 studies; risk ratio [RR], 6.76; 95% CI, 5.08-8.99) and a 3-fold increased risk of preterm birth in the cancer exposed group (16 studies; RR, 3.07; 95% CI, 2.37-3.98, with 14 of 16 studies identifying an increased risk). There was a 1.5-fold increase in the risk of placenta previa (2 studies; RR, 1.49; 95% CI, 1.10-2.01); however, only 2 studies examined this and one of these found no association. The risks for preterm rupture of membranes, gestational diabetes, and hypertensive disorders of pregnancy were not significantly different in the pooled analysis. The risk of composite pregnancy complications was doubled in pregnancies affected by PAC (21 studies; RR, 2.49; 95% CI, 2.03-3.06). As reported in Figure 2, there was a 42-fold increased risk of maternal death in those affected by PAC (3 studies; RR, 41.58; 95% CI, 20.38-84.83).

#### PAC and Intrapartum/Peripartum Complications

**Figure 3:**
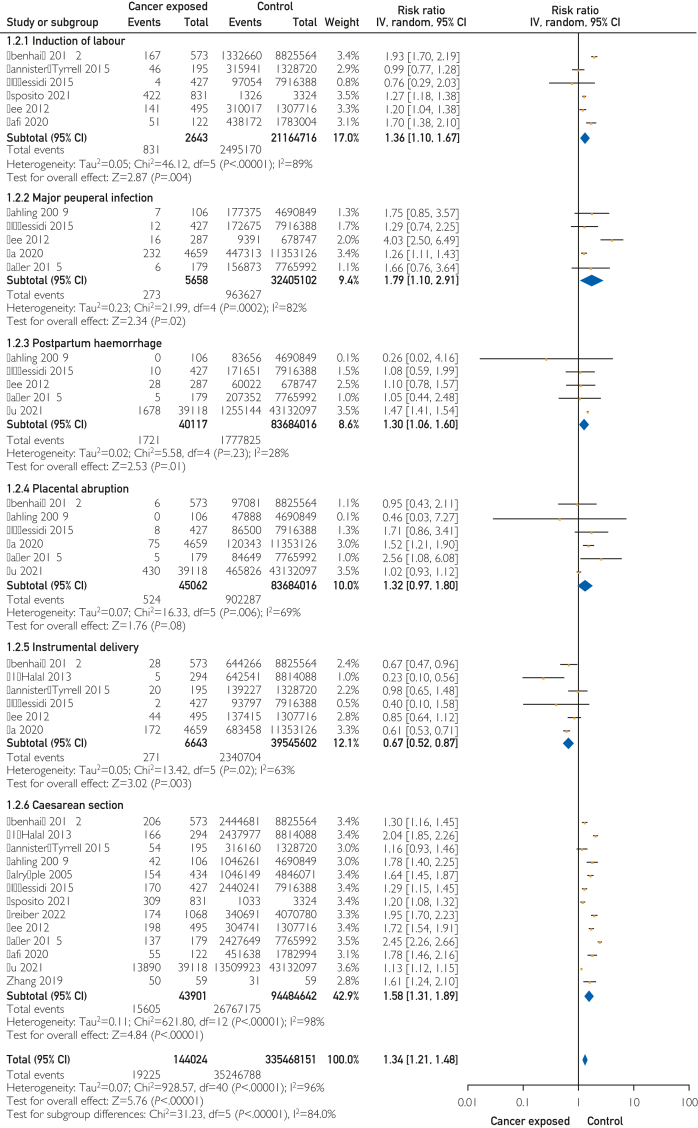
Pregnancy-associated cancer and intrapartum/peripartum complications.

Women with PAC had increased risk of intrapartum or peripartum complications (Figure 3). This included increased risk of induction of labor (6 studies; RR, 1.36; 95% CI, 1.10-1.67), postpartum hemorrhage (5 studies; RR, 1.30; 95% CI, 1.06-1.60), and major puerperal infection (5 studies; RR, 1.79; 95% CI, 1.10-2.91). Furthermore, the pooled analysis reported the risk of composite intrapartum or peripartum complications was increased in pregnancies affected by PAC (12 studies; RR, 1.34; 95% CI, 1.21-1.48). Regarding mode of birth (Figure 3), women with PAC were at increased risk of cesarean delivery (13 studies; RR, 1.58; 95% CI, 1.31-1.89) but were less likely to undergo an instrumental delivery compared with controls (6 studies; RR, 0.67; 95% CI, 0.52-0.87).

#### PAC and Fetal/Neonatal Complications

**Figure 4:**
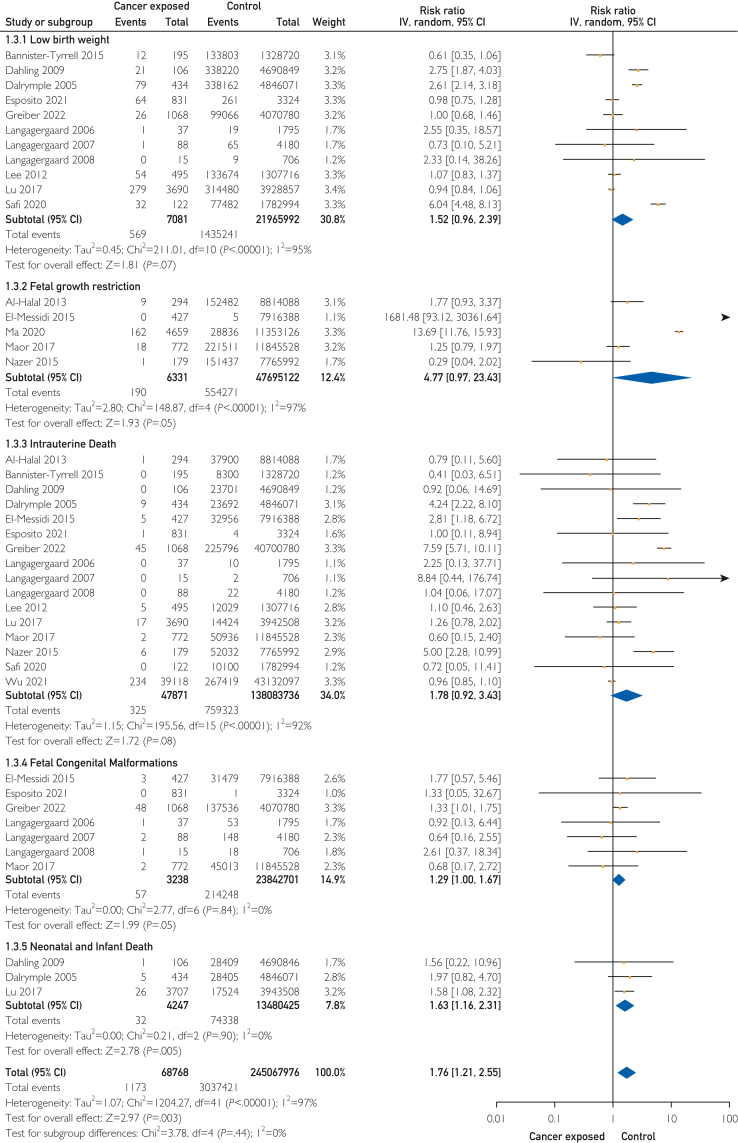
Pregnancy-associated cancer and fetal/neonatal complications.

There was no statistically significant increased risk for low-birth weight, intrauterine death, fetal growth restriction, or congenital malformations (Figure 4). There was a 1.7-fold increased risk of composite fetal/neonatal complications in pregnancies affected by PAC (17 studies; RR, 1.76; 95% CI, 1.21-2.55). We found a 1.6-fold risk of neonatal and infant death (3 studies; RR, 1.63; 95% CI, 1.16-2.31) in the cancer exposed group.

### Sensitivity Analyses

We performed a sensitivity analysis of studies on cancers diagnosed only during pregnancy and not in the 12 months after birth. There was minimal impact on the risk of adverse outcomes compared with those inclusive of cancers diagnosed up to 1 year after birth, except for a lower risk of major puerperal infection (pregnancy only: RR, 1.28; 95% CI, 1.14-1.45; vs pregnancy and postpartum: RR, 4.03; 95% CI, 2.50-6.49) (Supplemental Table 3, available online at http://www.mcpiqojournal.org).

We also conducted a sensitivity analysis by cancer type for breast, cervical, skin (inclusive of melanoma), and hematologic cancers (lymphoma and leukemia) (Supplemental Table 4, available online at http://www.mcpiqojournal.org). We were only able to assess a limited number of malignancies and outcomes owing to the lack of data, and hence, most of the results did not reach statistical significance. Breast cancer had the highest risk of preterm birth (RR, 5.62; 95% CI, 3.53-8.94) and low-birth weight (RR, 5.92; 95% CI, 4.41-7.95) compared with other cancers. Cervical cancer had the highest risk of cesarean delivery (RR, 1.50; 95% CI, 1.10-2.05), whereas hematologic cancer had the highest risk of intrauterine death (RR, 2.58; 95% CI, 1.12-5.92) compared with other cancer types (Supplemental Tables 4 and 5, available online at http://www.mcpiqojournal.org).

### Funnel Plots and Leave-1-Out Analysis

We performed funnel plots on composite outcomes, which included more than 10 publications that did not suggest publication bias. We also conducted a leave-1-out analysis on all outcomes and subgroups because heterogeneity was high in most outcomes and subgroups. However, no single source of heterogeneity was identified. There was low heterogeneity in studies with outcomes on venous thromboembolism, placenta previa, postpartum hemorrhage, congenital malformations, and neonatal and infant death.

## Discussion

Our meta-analysis of 22 studies included data from 59,190 pregnancies complicated by cancer with a control cohort of more than 70 million births. We found that women with PAC have double the risk of pregnancy, fetal, and neonatal complications. The most striking finding was a 42-fold increased risk of maternal mortality. Although up to 75% of cancers are diagnosed in the postpartum period,^1^ our meta-analysis did not report any difference in risk of adverse outcomes between antenatal and postnatal diagnosis, except for low-birth weight and blood transfusion. Our sensitivity analysis by cancer type found that breast cancer had the highest risk of preterm birth and low-birth weight. To our knowledge, this meta-analysis is the first to examine the impact of PAC on pregnancy outcomes.

Delaying motherhood is increasingly common worldwide, which may have contributed to the increase in incidence of pregnancies complicated by PAC over time. Although older women have higher risk of developing PAC^36^ and adverse pregnancy outcomes,^37^ a population-based cohort study of PAC by Lee et al^32^ found the increase in the incidence of PAC is attributable advanced maternal age in only 14% of cases. This suggests that age is only a minor contributing factor. The number of cases of PAC remains relatively small, affecting 1 in 1000 pregnancies.^1^ Hence, there is limited understanding of the risks of PAC, and previous reports of associations between PAC and birth outcomes have been varied.^38^ Current guidelines are limited by the mixed evidence base with only a few studies focusing on long-term prognosis.^39^, ^40^, ^41^ To further improve maternal and fetal outcomes, guidelines would benefit from more robust evidence on which to base their recommendations.

We reported a 42-fold increase in risk of maternal death, which equated to a rate of 372 per 100,000. This is much higher than that of the age-standardized mortality rate of 84.2 per 100,000 for the global estimate of all female cancers^42^ and suggests that pregnancy may increase the risk of cancer-related death. Regarding timing, maternal deaths occurred during the hospital admissions for delivery in all studies. Unfortunately, we do not have any data on the causes of death because these were not described in the studies. Therefore, we are unable to ascertain whether the increased risk of death is due to PAC itself, treatment for PAC, or comorbidities associated with PAC, such as thromboembolism or sepsis. Another possible reason for the increased risk of mortality may be that some women with PAC wish to delay cancer treatment owing to perceived potential fetal harm. A previous meta-analysis found that for every 4-week delay in curative treatment of cancer outside pregnancy, the risk of death increases.^43^ This was reported to be an increase of 6%-8% for surgical treatment, 9% for head and neck radiotherapy, and 13% for adjuvant systemic treatment for colorectal cancer. A 12-week delay of breast cancer treatment is associated with an increase in mortality risk of 17%.^43^ This pattern is reflected in our sensitivity analysis (Supplemental Table 3, available online at http://www.mcpiqojournal.org) in which poorer maternal outcomes were seen in 64% of those outcomes in which cancer had been diagnosed during pregnancy, compared with cancer diagnosed during both pregnancy and in the 12 months after delivery.

We found that women with PAC were at significantly increased risk of preterm birth and low-birth weight. Radiotherapy and chemotherapy may themselves adversely affect fetal growth through vascular and pulmonary impairment.^2^ A previous study found that children born to mothers exposed to chemotherapy during pregnancy have lower birth weight than those born to mothers not exposed to chemotherapy.^9^ Iatrogenic preterm birth may be planned to allow cancer treatment to start earlier in pregnancy,^2^ which may have contributed to the increased risk of low-birth weight in PAC. On the contrary, for breast cancer, anthracyclines are frequently given during the second and third trimesters of pregnancy and are not known to cause preterm birth.

Women with PAC were more likely to have a cesarean delivery than controls, although less likely to have an instrumental delivery. This suggests that when there is concern regarding the progress of labor or the well-being of the woman or her baby during labor, there may be a lower threshold for escalating to a cesarean section. However, the proportion of the cesarean deliveries that were planned vs emergency was not captured in the data. Cesarean delivery may enable earlier commencement of maternal cancer treatments, which are potentially toxic to the fetus.^9^^,^^23^^,^^30^ Furthermore, cesarean delivery may allow for sampling of tissue for histologic diagnosis^44^ or simultaneous surgical treatment of the cancer in selected scenarios.^45^ In certain cancers, such as cervical or vulval cancer, cesarean section is the preferred method of delivery owing to the risk of tumor seeding and/or birth canal obstruction.^41^ Nevertheless, an increased risk of cesarean delivery was observed across all studies, regardless of site of cancer.

We reported a 7-fold increased risk of venous thromboembolism in women with PAC. Pregnancy is a prothrombotic state, with physiologic increases in factors VII, VIII, and X and von Willebrand factor, along with a decrease in the protein S level.^45^ Cancer increases the risk of venous thrombotic events in varied ways. For example, venous thromboembolism usually develops in a valve sinus, where platelets and leukocytes tend to become trapped. Tumors can directly compress veins, resulting in venous stasis that encourages thrombosis. Tumors also release the procoagulant tissue factor that initiates the extrinsic pathway of the coagulation cascade. Inflammatory cytokines released from tumor cells and from local tissues in response to the tumor, such as tumor necrosis factor α and interleukins, can themselves impair the antithrombotic response through the production of fibrinolysis inhibitor PAI-1.^46^ Given the prothrombotic state of both pregnancy and malignancy, robust thromboprophylaxis protocols should be in place,^47^ and the diagnosis of venous thromboembolism should be actively considered in the assessment of an acutely unwell woman with PAC.

There is some evidence suggesting that breast cancer occurring during pregnancy differs distinctly from breast cancer occurring postpartum.^48^ With this in mind, we decided to use the more accepted definition of extending from 9 months before birth to 1 year postpartum. This definition includes the postpartum period because of the possibility of the cancer originating before birth^1^ but excludes a prolonged postpartum time frame in which the cancer is more likely to have occurred after birth. Interestingly, our sensitivity analysis on studies that focused on diagnosis during pregnancy only did not report significant differences in adverse outcomes to studies, including diagnosis within 1 year of giving birth.

This review and meta-analysis has multiple implications for practice. We have reported significant increases in risk of perinatal morbidity and mortality, which need to be communicated to women to enable well-informed shared decision-making. Furthermore, our meta-analysis will help health care professionals to plan appropriate birth methods for women with a diagnosis of PAC in the future. Delayed diagnosis has previously been cited as a contributing factor for increasing maternal morbidity for women with PAC.^49^ This suggests the importance of increased monitoring and specialist-led care in specialized centers for women with suspected PAC. Specialist management is also important owing to cancer treatments in pregnancy being associated with an increased risk of low-birth weight and prematurity.^50^ The psychosocial factors are also important considerations in the care of women with PAC. A systematic review found that women with PAC experience psychologic distress associated with concerns regarding the health of their neonate.^51^ Furthermore, the health care experience of these women are affected by communication within multidisciplinary care team.

A strength of this systematic review and meta-analysis is the large number of participants and cases of PAC drawn from large nationwide studies. Because this is the first meta-analysis in the field, our review may inform decision-making and future guidelines for PAC. The main limitation of this study is a function of the relative rarity of PAC. Because it is a rare diagnosis, only a few large-scale studies were available. Another limitation of this study is the high level of heterogeneity with many outcomes, although the leave-out analysis did not identify a single source of heterogeneity. Heterogeneity may also be due to the variation in study design and whether the study focused on a specific site or a range of cancers. Another limitation of this work is that it did not extend to the lifetime of the child and did not follow-up potential implications of PAC on the woman or child. A further limitation is the inability to systematically control for confounding. Many studies reported adjusted odds ratios, but owing to studies adjusting for different covariates, we were unable to account for confounding. Finally, most studies were of a low to moderate risk of bias.

## Conclusion

In conclusion, our analysis suggests that PAC diagnosed during 9 months before birth up to 1 year postpartum poses a significant increase in maternal and fetal morbidity and mortality. Further research is needed to evaluate the mechanisms that increase the risk of these adverse outcomes and how to best mitigate them.

## Potential Competing Interests

The authors report no competing interests.

## Supplemental Online Material

PRISMA_2020
