# Supplementary material for: Pregnancy-Associated Cancer: A Systematic Review and Meta-Analysis
Source: Mayo Clin Proc Innov Qual Outcomes. 2024 Mar 16;8(2):188–99. doi: 10.1016/j.mayocpiqo.2024.02.002 (PMC10957385; doi:10.1016/j.mayocpiqo.2024.02.002)
Supplement: Supplementary material [file mmc2.docx]

**Supplemental Material**

Supplemental Table 1. Search strategy for Medline.

| **#** | **Search term** |
| --- | --- |
| 1 | exp "PREGNANCY COMPLICATIONS, NEOPLASTIC"/ |
| 2 | ((pregnant OR pregnancy OR birth) AND (cancer* OR malignanc* OR neoplas* OR tumo?r*)).ti,ab |
| 3 | (1 OR 2) |
| 4 | exp "INFANT, LOW BIRTH WEIGHT"/ |
| 5 | (low birth weight*).ti,ab |
| 6 | exp "OBSTETRIC LABOR, PREMATURE"/ |
| 7 | (premature OR prematurity OR pre-term OR preterm).ti,ab |
| 8 | (small for gestational age).ti,ab |
| 9 | "EXTRACTION, OBSTETRICAL"/ |
| 10 | (assisted vaginal OR instrumental OR forceps).ti,ab |
| 11 | exp "CESAREAN SECTION"/ |
| 12 | (c?esarean section).ti,ab |
| 13 | (antepartum h?emorrhage).ti,ab |
| 14 | exp "PLACENTA DISEASES"/ |
| 15 | (placenta previa OR placenta praevia OR placental abruption).ti,ab |
| 16 | "POSTPARTUM HEMORRHAGE"/ |
| 17 | (postpartum h?emorrhage OR post-partum h?aemorrhage).ti,ab |
| 18 | exp "CONGENITAL ABNORMALITIES"/ |
| 19 | (congenital abnormalit*).ti,ab |
| 20 | "PRE-ECLAMPSIA"/ |
| 21 | (preeclampsia OR pre-eclampsia).ti,ab |
| 22 | exp HYPERTENSION/ |
| 23 | exp "HYPERTENSION, PREGNANCY-INDUCED"/ |
| 24 | (hypertension).ti,ab |
| 25 | exp "DIABETES, GESTATIONAL"/ |
| 26 | (gestational diabetes).ti,ab |
| 27 | "FETAL DISTRESS"/ |
| 28 | (f?etal distress).ti,ab |
| 29 | exp "FETAL DEATH"/ |
| 30 | (stillbirth OR stillborn).ti,ab |
| 31 | "FETAL GROWTH RETARDATION"/ |
| 32 | (growth restriction).ti,ab |
| 33 | (perinatal death OR perinatal mortality OR f*etal death).ti,ab |
| 34 | (venous thromboembolism).ti,ab |
| 35 | (maternal death) ti, ab |
| 36 | (4 OR 5 OR 6 OR 7 OR 8 OR 9 OR 10 OR 11 OR 12 OR 13 OR 14 OR 15 OR 16 OR 17 OR 18 OR 19 OR 20 OR 21 OR 22 OR 23 OR 24 OR 25 OR 26 OR 27 OR 28 OR 29 OR 30 OR 31 OR 32 OR 33 OR 34 OR 35) |
| 37 | (3 AND 36) |

Supplemental Table 2. Study characteristics of included studies.

| Authors | Year | Country | Study Design | Database used | Cancer type | Number of patients in cancer group | OCEBM Grade |
| --- | --- | --- | --- | --- | --- | --- | --- |
| Abdel-Hady et al.^54^ | 2012 | Egypt | Prospective Case-Control | Local database | Multiple | 118 women | IV |
| Abenhaim et al.^23^ | 2012 | Canada | Retrospective Cohort | U.S. National Inpatient Sample (HCUP-NIS) database | Breast | 573 women | III |
| Al-Halal et al.^55^ | 2013 | Canada | Retrospective Cohort | U.S. National Inpatient Sample (HCUP-NIS) database | Cervical | 294 deliveries | III |
| Bannister-Tyrell et al.^34^ | 2015 | Australia | Retrospective Cohort | New South Wales Population Databases | Melanoma | 577 women | III |
| Bleau et al.^53^ | 2016 | Canada | Retrospective Cohort | U.S. National Inpatient Sample (HCUP-NIS) database | Multiple | 2,826 deliveries | III |
| Dahling et al.^35^ | 2009 | USA | Retrospective Cohort | California Office of Planning and Development (OSHPD) database | Colorectal | 134 women | III |
| Dalrymple et al.^30^ | 2005 | USA | Retrospective Cohort | California Office of Planning and Development (OSHPD) database | Cervical | 434 women | III |
| El-Messidi et al.^52^ | 2015 | Canada | Retrospective Cohort | U.S. National Inpatient Sample (HCUP-NIS) database | Non-Hodgkin’s Lymphoma | 427 women | III |
| Esposito et al.^9^ | 2021 | Italy | Retrospective Cohort | Regional healthcare utilisation databases of Lombardy | Not specified | 831 women | III |
| Greiber et al.^25^ | 2021 | Denmark | Retrospective Cohort | Danish Health Registries | Multiple | 1,330 women | III |
| Greiber et al.^26^ | 2022 | Denmark | Retrospective Cohort | Danish Health Registries | Multiple | 1,068 pregnancies | III |
| Langergaard et al.^27^ | 2006 | Denmark | Retrospective Cohort | Danish Cancer Registry | Breast | 37 deliveries | III |
| Langergaard et al.^29^ | 2007 | Denmark | Retrospective Cohort | Danish Cancer Registry | Melanoma | 88 deliveries | III |
| Langergaard et al.^28^ | 2008 | Denmark | Retrospective Cohort | Danish Cancer Registry | Hodgkin’s disease | 15 deliveries | III |
| Lee et al.^32^ | 2012 | Australia | Retrospective Cohort | New South Wales Population Databases | Multiple | 1,798 women | III |
| Lu et al. ^33^ | 2017 | Sweden | Retrospective Cohort | Swedish Medical Birth Register | Multiple | 3,707 deliveries | III |
| Ma et al. ^3^ | 2020 | USA | Retrospective Cohort | U.S. National Inpatient Sample (HCUP-NIS) database | Multiple | 4,659 deliveries | III |
| Maor et al.^24^ | 2017 | Canada | Retrospective Cohort | U.S. National Inpatient Sample (HCUP-NIS) database | Breast | 772 deliveries | III |
| Nazer et al.^44^ | 2015 | Canada | Retrospective Cohort | U.S. National Inpatient Sample (HCUP-NIS) database | Ovarian | 179 women | III |
| Safi et al.^31^ | 2021 | Australia | Retrospective Cohort | New South Wales Population Databases | Breast | 122 women | III |
| Wu et al.^2^ | 2021 | UK | Retrospective Cohort | U.S. National Inpatient Sample (HCUP-NIS) database | Multiple | 39,118 deliveries | III |
| Zhang et al.^10^ | 2019 | China | Prospective Cohort | Local database | Multiple | 83 women | III |

HCUP-NIS, Healthcare Cost and Utilization Project – Nationwide Inpatient Sample

Supplemental Table 3. Sensitivity analysis on cancers diagnosed during pregnancy only, excluding cancers diagnosed postpartum.

| **Outcome** | **Cancer diagnosed during pregnancy and up to 1 year postpartum** | **Cancer diagnosed during pregnancy only** |
| --- | --- | --- |
| Premature rupture of membranes | --- | 1.51 [0.59, 3.88], n=6 |
| Preterm birth | 2.90 [2.06, 4.07], n=7 | 3.20 [2.08, 4.93], n=8 |
| Venous thromboembolism | 7.92 [4.70, 13.35], n=2 | 6.32 [4.50, 8.88], n=4 |
| Gestational diabetes | 1.18 [0.86, 1.62], n=1 | 0.95 [0.65, 1.39], n=3 |
| Placenta praevia | --- | 1.49 [1.10, 2.01], n=2 |
| Hypertensive disorders of pregnancy | 1.11 [0.93, 1.33], n=1 | 1.11 [0.86, 1.43], n=6 |
| Maternal death | --- | 41.588 [20.38, 84.83], n=3 |
| Instrumental delivery | 0.89 [0.70, 1.12], n=2 | 0.56 [0.42, 0.76], n=4 |
| Caesarean section | 1.53 [1.27, 1.83], n=3 | 1.60 [1.28, 2.00], n=10 |
| Induction of labour | 1.13 [0.94, 1.34], n=2 | 1.52 [1.14, 2.03], n=4 |
| Major puerperal infection | 4.03 [2.50, 6.49], n=1 | 1.28 [1.14, 1.45], n=4 |
| Blood transfusion | 6.21 [3.13, 12.34], n=1 | 6.19 [2.97, 12.92], n=5 |
| Postpartum haemorrhage | 1.10 [0.78, 1.57], n=1 | 1.45 [1.31, 1.61], n=5 |
| Placental abruption | --- | 1.32 [0.97, 1.80], n=6 |
| Low birth weight | 1.20 [0.71, 2.02], n=7 | 2.01 [0.80, 5.04], n=4 |
| Fetal growth restriction | --- | 4.77 [0.97, 23.43], n=5 |
| Intrauterine death | 1.78 [0.92-3.46], n=7 | 1.73 [0.65, 4.58], n=9 |
| Fetal congenital malformations | 1.00 [0.38, 2.64], n=3 | 1.32 [1.01, 1.72], n=4 |

Data presented as risk ratio [95% confidence interval]. n=number of studies.

Supplemental Table 4. Sensitivity analysis on sites of cancer.

| **Outcome** | **Breast cancer** | **Cervical cancer** | **Skin cancer** | **Haematological cancers** |
| --- | --- | --- | --- | --- |
| Premature Rupture of Membranes | 2.43 [1.59, 3.70], n=1 | 0.89 [0.48, 1.64], n=1 | --- | 348.99 [21.30, 5717.35], n=1 |
| Preterm birth | 5.62 [3.53, 8.94], n=3 | 2.65 [1.60, 4.38], n=2 | 3.17 [0.21, 46.90], n=2 | 0.89 [0.09, 8.67], n=2 |
| Venous thromboembolism | --- | 4.00 [1.01, 15.83], n=1 | --- | 3.25 [0.46, 22.99], n=1 |
| Gestational diabetes | 1.22 [0.70, 2.13], n=2 | --- | --- | 0.79 [0.50, 1.25], n=1 |
| Placenta praevia | --- | --- | --- | 0.78 [0.20, 3.13], n=1 |
| Hypertensive disorders of pregnancy | 0.68 [0.44, 1.06], n=2 | --- | --- | 1.59 [1.10, 2.29], n=1 |
| Maternal death | --- | --- | --- | 84.91 [27.42, 262.92], n=1 |
| Instrumental delivery | 0.67 [0.47, 0.96], n=1 | 0.23 [0.10, 0.56], n=1 | 0.98 [0.65, 1.48], n=1 | 0.40 [0.10, 1.58], n=1 |
| Caesarean delivery | 1.50 [1.10, 2.05], n=2 | 1.84 [1.49, 2.27], n=2 | 1.16 [0.93, 1.46], n=1 | 1.29 [1.15, 1.45], n=1 |
| Induction of labour | 1.86 [1.67, 2.08], n=2 | --- | 0.99 [0.77, 1.28], n=1 | 0.76 [0.29, 2.03], n=1 |
| Major puerperal infection | --- | --- | --- | 1.29 [0.74, 2.25], n=1 |
| Blood transfusion | --- | 11.92 [3.62, 39.29], n=2 | --- | 2.63 [2.11, 3.27], n=1 |
| Postpartum haemorrhage | --- | --- | --- | 1.08 [0.59, 1.99], n=1 |
| Placental abruption | 0.95 [0.43, 2.11], n=1 | --- | --- | 1.71 [0.86, 3.41], n=1 |
| Low birth weight | 5.92 [4.41, 7.95], n=2 | 2.61 [2.14, 3.18], n=1 | 0.62 [0.37, 1.05], n=2 | 2.33 [0.14, 38.26], n=1 |
| Fetal growth restriction | --- | 1.77 [0.93, 3.37], n=1 | --- | 1681.48 [93.12, 30361.64], n=1 |
| Intrauterine death | 0.77 [0.25, 2.38], n=3 | 2.39 [0.50, 11.38], n=2 | 1.80 [0.09, 36.50] n=2 | 2.58 [1.12, 5.92], n=2 |
| Fetal congenital malformations | 0.75 [0.24, 2.33], n=1 | --- | 0.64 [0.16, 2.55], n=1 | 1.95 [0.73, 5.17], n=2 |

Data presented as risk ratio [95% confidence interval]. n=number of studies.

Supplemental Table 5. Sensitivity analysis of haematological versus non-haematological malignancies

| **Outcome** | **Haematological** | **Non-haematological** |
| --- | --- | --- |
| Premature rupture of membranes | 348.99 [21.30, 5717.35], n=1 | 1.49 [0.86, 2.59], n=4 |
| Preterm birth | 0.89 [0.09, 8.67], n=2 | 3.45 [2.41, 4.93], n=9 |
| Venous thromboembolism | 3.25 [0.46, 22.99], n=1 | 7.30 [3.05, 17.48], n=2 |
| Gestational diabetes | 0.79 [0.50, 1.25], n=1 | 1.22 [0.70, 2.13], n=2 |
| Placenta praevia | 0.78 [0.20, 3.13], n=1 | --- |
| Hypertensive disorders of pregnancy | 1.59 [1.10, 2.29], n=1 | 0.81 [0.58, 1.14], n=4 |
| Maternal death | 84.91 [27.42, 262.92], n=1 | 55.84 [7.90, 394.72], n=1 |
| Instrumental delivery | 0.40 [0.10, 1.58], n=1 | 0.60 [0.33, 1.10], n=3 |
| Caesarean section | 1.29 [1.15, 1.45], n=1 | 1.70 [1.38, 2.10], n=7 |
| Induction of labour | 0.76 [0.29, 2.03], n=1 | 1.50 [1.05, 2.16], n=3 |
| Major puerperal infection | 1.29 [0.74, 2.25], n=1 | 1.71 [1.00, 2.90], n=2 |
| Blood transfusion | 2.63 [2.11, 3.27], n=1 | 8.70 [3.66, 20.68], n=4 |
| Postpartum haemorrhage | 1.08 [0.59, 1.99], n=1 | 0.92 [0.41, 2.11], n=2 |
| Placental abruption | 1.71 [0.86, 3.41], n=1 | 1.39 [0.60, 3.22], n=3 |
| Low birth weight | 2.33 [0.14, 38.26], n=1 | 2.17 [1.15, 4.10], n=6 |
| Fetal growth restriction | 1681.48 [93.12, 30361.64], n=1 | 1.29 [0.75, 2.21], n=3 |
| Intrauterine death | 2.58 [1.12, 5.92], n=2 | 2.07 [1.02, 4.23], n=9 |
| Foetal congenital anomaly | 1.95 [0.73, 5.17], n=2 | 0.71 [0.29, 1.69], n=3 |

Data presented as risk ratio [95% confidence interval]. n=number of studies.

| Authors | 1 | 2 | 3 | 4 | 5 | 6 | 7 | 8 | 9 | 10 | 11 | 12 | 13 | 14 | 15 | 16 | 17 | 18 |
| --- | --- | --- | --- | --- | --- | --- | --- | --- | --- | --- | --- | --- | --- | --- | --- | --- | --- | --- |
| Abdel-Hady et al. 2012 ^54^ |  |  |  |  |  |  |  |  |  |  |  |  |  |  |  |  |  |  |
| Abenhaim et al. 2012 ^23^ |  |  |  |  |  |  |  |  |  |  |  |  |  |  |  |  |  |  |
| Al-Halal et a. 2013 ^55^ |  |  |  |  |  |  |  |  |  |  |  |  |  |  |  |  |  |  |
| Bannister-Tyrell et al. 2015 ^34^ |  |  |  |  |  |  |  |  |  |  |  |  |  |  |  |  |  |  |
| Bleau et al. 2016 ^53^ |  |  |  |  |  |  |  |  |  |  |  |  |  |  |  |  |  |  |
| Dahling et al.2009 ^35^ |  |  |  |  |  |  |  |  |  |  |  |  |  |  |  |  |  |  |
| Dalrymple et al. 2005 ^30^ |  |  |  |  |  |  |  |  |  |  |  |  |  |  |  |  |  |  |
| El-Messidi et al 2015 ^52^ |  |  |  |  |  |  |  |  |  |  |  |  |  |  |  |  |  |  |
| Esposito et al. 2021 ^9^ |  |  |  |  |  |  |  |  |  |  |  |  |  |  |  |  |  |  |
| Greiber et al. 2021 ^25^ |  |  |  |  |  |  |  |  |  |  |  |  |  |  |  |  |  |  |
| Greiber et al 2022 ^26^ |  |  |  |  |  |  |  |  |  |  |  |  |  |  |  |  |  |  |
| Langergaard et al. 2006 ^27^ |  |  |  |  |  |  |  |  |  |  |  |  |  |  |  |  |  |  |
| Langergaard et al. 2007 ^29^ |  |  |  |  |  |  |  |  |  |  |  |  |  |  |  |  |  |  |
| Langergaard et al. 2008 ^28^ |  |  |  |  |  |  |  |  |  |  |  |  |  |  |  |  |  |  |
| Lee et al. 2012 ^32^ |  |  |  |  |  |  |  |  |  |  |  |  |  |  |  |  |  |  |
| Lu et al. 2017 ^33^ |  |  |  |  |  |  |  |  |  |  |  |  |  |  |  |  |  |  |
| Ma et al. 2018 ^3^ |  |  |  |  |  |  |  |  |  |  |  |  |  |  |  |  |  |  |
| Maor et al. 2017 ^24^ |  |  |  |  |  |  |  |  |  |  |  |  |  |  |  |  |  |  |
| Nazer et al.2015 ^44^ |  |  |  |  |  |  |  |  |  |  |  |  |  |  |  |  |  |  |
| Safi et al. 2021 ^31^ |  |  |  |  |  |  |  |  |  |  |  |  |  |  |  |  |  |  |
| Wu et al. 2021 ^2^ |  |  |  |  |  |  |  |  |  |  |  |  |  |  |  |  |  |  |
| Zhang et al. 2019 ^10^ |  |  |  |  |  |  |  |  |  |  |  |  |  |  |  |  |  |  |
|  |  |  |  |  |  |  |  |  |  |  |  |  |  |  |  |  |  |  |
| Note: Green = Yes (low risk of bias); Red = No (high risk of bias); Yellow = unclear (unclear risk of bias); Gray = Not applicable.  1. Were participants a representative sample selected from a relevant patient population?  2. Were the inclusion/exclusion criteria of participants clearly described?  3. Were participants entering the study at a similar point in their disease progression?  4. Was selection of patients consecutive?  5. Was data collection undertaken prospectively?  6. Were the groups comparable on demographic characteristics and clinical features?  7. Was the intervention (and comparison) clearly defined?  8. Was the intervention undertaken by someone experienced at performing the procedure?  9. Were the staff, place, and facilities where the patients were treated appropriate for performing the procedure?  10. Were any of the important outcomes considered (ie, on clinical effectiveness, cost-effectiveness, or learning curves)?  11. Were objective outcome measures used, including satisfaction scale?  12. Was the assessment of main outcomes blind?  13. Was follow-up long enough (≥1 year) to detect important effects on outcomes of interest?  14. Was information provided on non-respondents, dropouts?  15. Were the characteristics of withdrawals/dropouts similar to those that completed the study and therefore unlikely to cause bias?  16. Was length of follow-up similar between comparison groups.  17. Were the important prognostic factors identified?  18. Were the analyses adjusted for confounding factors? | | | | | | | | | | | | | | | | | | |

Supplemental Figure 1. Bias analysis.
